# Structural Equation Modeling and Whole-Genome Scans Uncover Chromosome Regions and Enriched Pathways for Carcass and Meat Quality in Beef

**DOI:** 10.3389/fgene.2018.00532

**Published:** 2018-11-13

**Authors:** Joel D. Leal-Gutiérrez, Fernanda M. Rezende, Mauricio A. Elzo, Dwain Johnson, Francisco Peñagaricano, Raluca G. Mateescu

**Affiliations:** ^1^Department of Animal Sciences, University of Florida, Gainesville, FL, United States; ^2^Faculdade de Medicina Veterinária, Universidade Federal de Uberlândia, Uberlândia, Brazil; ^3^University of Florida Genetics Institute, University of Florida, Gainesville, FL, United States

**Keywords:** cellular compartmentalization, cellular differentiation, cellular proliferation, fat deposition, postmortem proteolysis

## Abstract

Structural equation models involving latent variables are useful tools for formulating hypothesized models defined by theoretical variables and causal links between these variables. The objectives of this study were: (1) to identify latent variables underlying carcass and meat quality traits and (2) to perform whole-genome scans for these latent variables in order to identify genomic regions and individual genes with both direct and indirect effects. A total of 726 steers from an Angus-Brahman multibreed population with records for 22 phenotypes were used. A total of 480 animals were genotyped with the GGP Bovine F-250. The single-step genomic best linear unbiased prediction method was used to estimate the amount of genetic variance explained for each latent variable by chromosome regions of 20 adjacent SNP-windows across the genome. Three types of genetic effects were considered: (1) direct effects on a single latent phenotype; (2) direct effects on two latent phenotypes simultaneously; and (3) indirect effects. The final structural model included carcass quality as an independent latent variable and meat quality as a dependent latent variable. Carcass quality was defined by quality grade, fat over the ribeye and marbling, while the meat quality was described by juiciness, tenderness and connective tissue, all of them measured through a taste panel. From 571 associated genomic regions (643 genes), each one explaining at least 0.05% of the additive variance, 159 regions (179 genes) were associated with carcass quality, 106 regions (114 genes) were associated with both carcass and meat quality, 242 regions (266 genes) were associated with meat quality, and 64 regions (84 genes) were associated with carcass quality, having an indirect effect on meat quality. Three biological mechanisms emerged from these findings: postmortem proteolysis of structural proteins and cellular compartmentalization, cellular proliferation and differentiation of adipocytes, and fat deposition.

## Introduction

Many economically important characteristics in livestock could be described by a set of individual traits which often are themselves quantitative in nature. Certain relationships also exist between these traits given that they measure some common attributes of the system. Deriving a network of unobserved latent variables from the individual measured traits can be achieved by applying structural equation (SE) modeling (Schumacker et al., 2010; Rosa et al., 2011). Latent variables are defined as variables that are not directly measurable but can be characterized from several observed phenotypes. Latent variable modeling allows to investigate complex phenomena, such as meat quality, reducing at the same time data dimensionality because many phenotypes are combined to represent few underlying concepts of interest. The SE analysis determines if the theoretical model is supported by the sample data, and the theoretical model can be modified and retested until a model fitting the sample data is acquired (Schumacker et al., 2010).

In this study, we hypothesized that carcass composition is weight-dependent and influences meat quality traits. Carcass composition is weight-dependent, where fat is deposited chronologically as intermuscular, internal, subcutaneous, and intramuscular fat (Robelin et al., 1977; del Campo et al., 2008). Therefore, growth rate can be considered an important factor with direct effect on carcass composition and a fast growth rate resulting from a high plane of nutrition would lead to animals fattening earlier (Lawrie, 1998; del Campo et al., 2008). Low to medium genetic and phenotypic correlations among growth, carcass quality and meat quality traits, such as hot carcass weight and fat over the ribeye, hot carcass weight and shear force, marbling and tenderness have been reported in different breeds (Johnston et al., 2003; Reverter et al., 2003; Mateescu et al., 2015; Elzo et al., 2017).

The SE modeling analysis combined with the whole-genome scan approach can provide a powerful approach to uncovering not only direct and indirect genomic region contributions to variation in latent variables, but also identifying genetic regions with pleiotropic effects responsible for observed genetic correlations.

The objectives of this study were to: (1) identify a theoretical model involving beef growth, carcass quality and meat quality that closely fits the variance-covariance structure present in the sample phenotypic data and (2) perform whole-genome scans for the latent variables constructed in the SE analysis to identify genomic regions with direct and indirect effects.

## Materials and Methods

### Cattle Population and Phenotypic Data

The research protocol was approved by the University of Florida Institutional Animal Care and Use Committee number 201003744. Records from 726 steers born between 1990 and 2015 that belong to the Angus-Brahman multibreed herd from University of Florida (Elzo et al., 2015a,b, 2016; Leal-Gutiérrez et al., 2018), and a four-generation pedigree with 1,122 animals were used in this study. A total of 22 phenotypes were utilized to construct three latent variables: growth, carcass quality, and meat quality.

Birth weight, 205-day adjusted weaning weight, 365-day adjusted yearling weight and 525-day adjusted slaughter weight were used to calculate daily gain from birth to weaning, weaning to yearling and yearling to slaughter.

Steers were transported to a commercial packing plant when FOR reached approximately 1.27 cm. The average slaughter weight was 542.9 ± 59.6 kg at 18.22 ± 1.51 months. Steers were harvested using established USDA-FSIS procedures. Recorded carcass traits were hot carcass weight, dressing percentage, FOR, ribeye area, kidney, pelvic and heart fat (KPH) percentage, MARB (100 to 199 = practically devoid, 200 to 299 = traces, 300 to 399 = slight, 400 to 499 = small, 500 to 599 = modest, 600 to 699 = moderate, 700 to 799 = slightly abundant, 800 to 899 = moderately abundant, and 900 to 999 = abundant), yield grade and quality grade (QG) measurements were recorded. Lean maturity, bone maturity, and overall maturity were also determined but they were excluded from the analysis because they were highly collinear with other traits in this dataset.

One 2.54 cm steak from the *longissimus dorsi* muscle at the 12^th^/13^th^ rib interface was sampled from each animal. Steaks were individually identified, vacuum packaged and transported to the University of Florida Meat Processing Center (Gainesville, FL, United States), aged for 14 days at 1 to 4°C, and, then stored at -20°C. Prior to the sensory panel, each steak was allowed to thaw at 4°C for 24 h and cooked to an internal temperature of 71°C in an open-hearth grill. Connective tissue (CT), tenderness (TD), juiciness (JC), and flavor were assessed by sensory panel accordingly to the American Meat Science Association Sensory Guidelines (Belk et al., 2015). Six animals were evaluated by a sensory panel of 8–11 trained members each session. Each panelist scored two 1 cm × 2.54 cm samples from each steak for CT, TD, JC, and flavor on a scale from 1 to 8 (1 being the most undesirable and 8 being the most desirable; Wright et al., 2018).

### Structural Equation Analysis

The birth weight, 205-day adjusted weaning weight, 365-day adjusted yearling weight, 525-day adjusted slaughter weight, daily gain from birth to weaning, daily gain from weaning to yearling and daily gain from yearling to slaughter were corrected for year of birth. All the other traits were adjusted for year of birth and slaughter age. Residuals from a GLM model were used in the subsequent analyses. Phenotypic correlations between the observed variables included in the SE analysis are presented in Supplementary Figure S1.

The SE analyses building process started with model specification, identification and estimation, followed by model testing and modification (Schumacker et al., 2010).

The R package lavaan (R Core Team, 2008; Rosseel, 2012) was used to model latent variables underlying growth, carcass, and meat quality traits. The equations of the structural model were the following:

$$\begin{array}{l} \text{x}=\lambda_{\text{x}}\xi +\delta \\ \text{y}=\lambda_{\text{y}}\text{η}+\text{ε}\\ \text{η}=\text{Bη}+\Gamma \xi +\zeta \end{array}$$

where x and y are the vectors of adjusted phenotypic traits; η and ξ are the vectors of endogenous and exogenous latent constructs, respectively; elements of λ are factor loadings relating latent variables to the observed phenotypes (indicator traits); entries of B and Γ are path (causal) coefficients between latent variables; δ, 𝜀, and ς are vectors of residual terms (Rosa et al., 2011; Peñagaricano et al., 2015).

Model identification was performed using the number of observed variables included in the final structural model (p), the number of estimated parameters (q) and the following formula:

df=((p×(p+1))/2)−q

When df ≥ 1, the model was determined to be overidentified (Schumacker et al., 2010).

The model was fitted using the maximum likelihood estimation with robust standard errors and a mean- and variance-adjusted test statistic (Peñagaricano et al., 2015) and unstandardized loadings were estimated for each latent variable construct.

The structural model fitness, representing the difference between the observed and model-implied variance–covariance matrices, was assessed using the χ^2^ goodness of fit test and several alternative fit indices (Schumacker et al., 2010), including: goodness of fit index, adjusted goodness of fit index, root mean residual, comparative fit index, and root mean square error of approximation (Gefen and Straub, 2000). If the implied theoretical model was significantly different than the observed, the structural model was modified and retested (Schumacker et al., 2010). Significance of each path specific hypothesis and the direction of loading factors were tested.

The semPlot (Epskamp, 2017) and qgraph (Epskamp et al., 2012) R packages were used for graphical representation of the final model. The accepted model was tested for bias by permutation using the bootstrapLavaan function from the lavaan package with 5000 bootstrap draws.

### Whole-Genome Scan Analysis

Genomic DNA was extracted from blood samples using the DNeasy Blood & Tissue kit (Qiagen, Valencia, CA, United States) and stored at -20°C. All animals were genotyped with the GGP Bovine F-250 SNP chip (GeneSeek, Inc., Lincoln, NE, United States). The genotype data is available in the EVA website, accession number PRJEB24746. Out of 221,077 SNPs only 112,267 SNPs were retained for the Whole-genome scan association analysis after excluding non-polymorphic SNPs, markers with minor allele frequency lower than 0.05, and SNPs with calling rate less than 0.9.

A four-generation pedigree-based relationship matrix (A) was constructed using 612 steers with phenotypes that were progeny of 143 sires. The single-step genomic best linear unbiased prediction (ssGBLUP) approach implemented in the BLUPf90 family of programs (Misztal, 2002; Wang et al., 2012) was used to estimate the amount of genetic variance explained by chromosome regions of 20 adjacent SNP-windows for each latent variable. The ssGBLUP combines pedigree, phenotypic, and genotypic information and utilizes the relationship matrix H which combines pedigree and genotypic information (Aguilar et al., 2010). Single-trait models for the latent variables were analyzed with BLUPf90. The exogenous latent variable was used as covariate for the endogenous latent variable to correct for causality in order to identify direct and indirect effects on the endogenous latent variable (Li et al., 2006). POSTGSf90 was used to estimate the genetic variance explained by each chromosome region.

**FIGURE 1:**
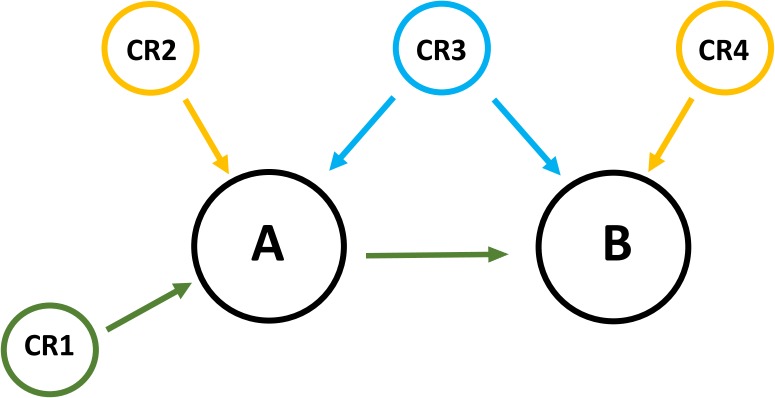
Three types of genetic effects were considered. (1) The chromosome region CR1 has direct effects on A and an indirect effect on B giving the causality relationship between A and B. The effect of the region CR1 on B disappears after using the latent phenotype A as a covariable in the association between CR1 and B; (2) The chromosome regions CR2 and CR4 have direct genetic effects on the latent phenotypes A and B respectively; (3) The chromosome region CR3 has a direct effect on A and B. The effect of the region CR3 remains even after using the latent phenotype A as a covariable in the association between CR3 and B.

Three types of genetic effects were considered: (1) direct effects on a single latent phenotype; (2) direct effects on two latent phenotypes simultaneously; and (3) indirect effects (Figure 1). Chromosome regions able to explain more than 0.15% of the additive genetic variance were determined as associated with the latent variable of interest.

### Functional Annotation Clustering Analysis

Chromosome regions explaining at least 0.05% of the additive variance in carcass quality and meat quality latent variables were considered for this analysis, and genes located inside these regions were identified using the Biomart tool from the Ensembl genome browser (Zerbino et al., 2018). All genes inside the associated chromosome regions were included in a functional classification analysis using DAVID Bioinformatic Resources 6.8 server (Huang et al., 2009a,b).

## Results and Discussion

Table 1 presents the mean and standard deviation for the phenotypes used in the construction of the latent variables. Similar values have been reported for these traits in Brahman and Brahman-influenced populations (Riley et al., 2003; Smith et al., 2007).

**Table 1:** Mean and SD for the observed phenotypes used to construct and measure the growth, carcass quality, and meat quality latent variables in *longissimus dorsi* muscle.

Trait	Mean	*SD*
Birth weight (kg)	34.60	6.16
205-day adjusted weaning weight (kg)	226.80	28.64
365-day adjusted yearling weight (kg)	339.38	54.64
525-day adjusted slaughter weight (kg)	523.80	71.50
Daily gain from birth to weaning (kg/day)	0.94	0.13
Daily gain from weaning to yearling (kg/day)	0.70	0.27
Daily gain from yearling to slaughter (kg/day)	1.15	0.27
Hot carcass weight (kg)	332.05	42.82
Dressing percentage	62.04	2.99
Lean maturity	147.49	11.94
Bone maturity	147.69	11.09
MARB	400.58	94.27
FOR (in)	1.32	0.48
Ribeye area (in^2^)	31.01	4.17
KPH (%)	1.98	0.98
Yield grade	3.20	0.70
QG	596.76	72.34
JC	5.24	0.72
Flavor	5.60	0.49
TD	5.41	0.89
CT	5.92	0.89

*MARB, marbling; FOR, fat over ribeye; KPH, kidney, pelvic and heart fat; QG, quality grade; JC, juiciness; TD, tenderness; CT, connective tissue*.

### Structural Equation Model for Carcass and Meat Quality Traits

**FIGURE 2:**
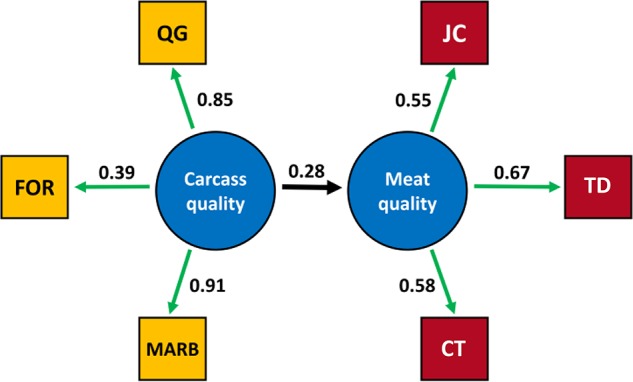
Relationship between observed variables and the latent variables carcass quality and meat quality in *longissimus dorsi* muscle in the final SE model. The estimation of unstandardized loadings are presented. The estimated structural coefficient between the carcass quality and meat quality latent variables was 0.28. MARB, marbling; FOR, fat over ribeye; QG, quality grade; JC, juiciness; TD, tenderness; CT, connective tissue.

The final structural model included carcass quality as an independent latent variable and meat quality as a dependent latent variable where carcass quality was measured by QG, FOR and MARB, and meat quality measured by JC, TD, and CT (Figure 2). The final structural model was overidentified (10 df) and the variance-covariance matrix for the observed variables included in the final structural model is presented in Supplementary Table S1. The final structural model fitted the sample data (χ^2^= 0.06, Supplementary Table S2), and the goodness of fit index (0.99), adjusted goodness of fit index (0.98), root mean residual (0.01), comparative fit index (0.99), and root mean square error of approximation (0.04) parameters were satisfied. The structural model based on theoretical knowledge did satisfy the variance-covariance structure present in the sample data with less than 4% of the draws in the permutation assessment not fitting the structural model. No latent variable for growth or observed growth related variables fitted the structural model.

The MARB variable had the strongest relationship with the carcass quality latent variable, and the observed TD variable accounted for the highest proportion of variability in the meat quality latent variable based on the estimation on the unstandardized loadings. The structural coefficients determine the extent to which a latent variable vary linearly with other latent variables in the model (Gefen and Straub, 2000), and a 0.28 unit change in the meat quality latent variable was estimated for one unit change in the carcass quality latent variable (Supplementary Table S2). The positive direction was expected given that the carcass quality construct reflects mainly fat deposition, and fat related traits have been associated with better meat quality (O’Connor et al., 1997; Smith et al., 2007).

The direction of the loading factors agrees with the theoretical model where FOR, QG, and MARB are positively related to carcass quality. These three observed phenotypes are positively correlated being FOR associated with MARB development and therefore QG. The observed variables JC, TD, and CT are also positively related with meat quality, indicating that consumer perception of high quality is determined by high JC, high TD and lower perception of connective particles during the eating experience (high CT score).

Both, positive and negative relationships between growth related traits and carcass/meat quality have been reported (Lawrie, 1998; Castro Bulle et al., 2007; del Campo et al., 2008) and this relationship is mostly due to differences in growth curves between frame types (Menchaca et al., 1996). Differences in daily gain exist even within frame type and these could have an effect on carcass and meat quality. In late maturing animals a strong negative relationship between growth, carcass and meat quality parameters was suggested, where steers with higher daily gain showed a lack of maturity at slaughter with a direct negative effect on MARB, FOR, KPH, ribeye area, and meat quality (Aass, 1996; Castro Bulle et al., 2007). The present population is characterized by mostly frame 1 animals (early maturing, short legs and body, lack of rapid growth potential but able to have good muscle growth) with a weak phenotypic correlation between growth and meat quality traits (Smith et al., 2007; Tonussi et al., 2015). The estimated heritabilities for the latent phenotypes carcass quality and taste quality were 0.46 ± 0.10 and 0.36 ± 0.10, respectively, and their genetic correlation was 0.87 ± 0.09. Genetic parameters for individual growth, carcass quality, and meat palatability traits have been estimated in the present population and reported previously (Elzo et al., 2012, 2013, 2015a, 2016, 2017).

### Whole-Genome Scan for Carcass and Meat Quality Latent Variables

**FIGURE 3:**
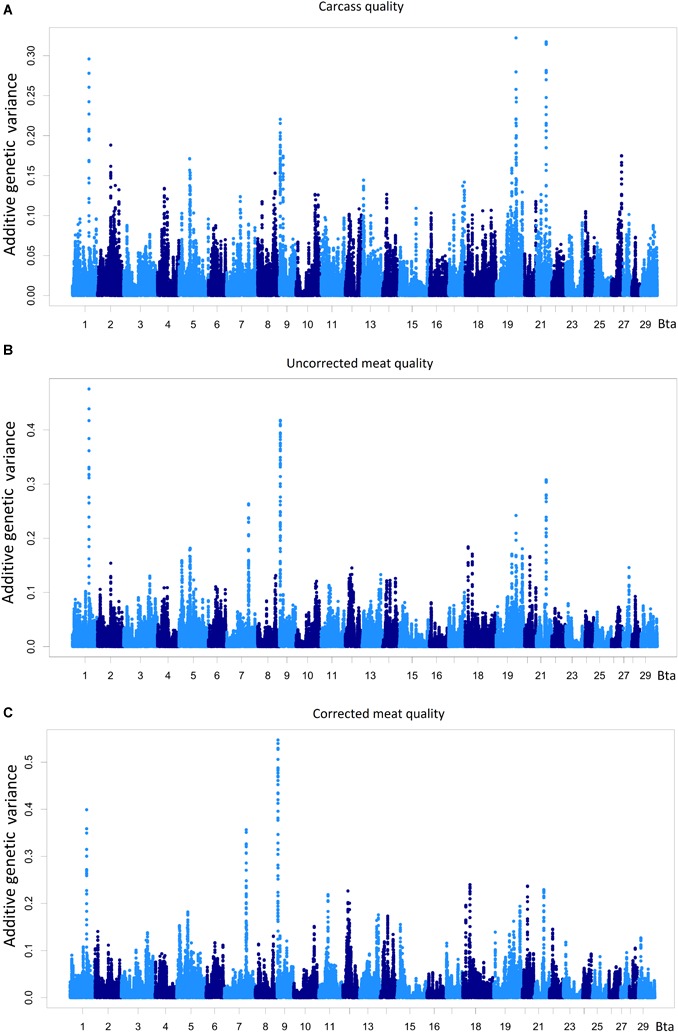
Percentage of the additive genetic variance explained by chromosome regions of 20 adjacent SNP-windows for the phenotypes carcass quality **(A)**, meat quality **(B)**, and meat quality corrected by carcass **(C)**. All the measurements were taken on the *longissimus dorsi* muscle. The total amount of additive genetic variance present in carcass quality was 29.17 and 41.33% for meat quality.

Data dimensionality reduction in the SE analysis using latent variable constructions (Peñagaricano et al., 2015) is advantageous for whole-genome scan assessment of highly correlated phenotypes. The proportion of additive genetic variance explained by chromosome regions of 20 adjacent SNP-window for the latent variables carcass quality, meat quality, and meat quality with carcass quality as covariate is shown in Figure 3 and Supplementary Table S3.

A total of 571 regions that explained more than 0.05% of the additive variance present in the latent variables were identified, and 82 of these regions were located on BTA12 and BTA19. One hundred and fifty-nine chromosome regions were associated with the carcass quality latent variable, and 242 chromosome regions had a direct effect on the meat quality latent variable. A total of 106 chromosome regions were associated with carcass, meat quality and meat quality with carcass as a covariate, and 64 regions were associated with carcass quality, having an indirect effect on meat quality. The QTLs harbored by these 170 chromosome regions could be contributing to the genetic correlation observed between the carcass quality and meat quality latent variables in the present population. These QTLs may also help explain genetic correlations between the component traits of these latent variables and other carcass and meat quality traits reported in numerous studies and diverse populations (O’Connor et al., 1997; Reverter et al., 2003; Riley et al., 2003; Smith et al., 2007).

#### Genomic Regions Associated With the Carcass Quality Latent Variable

**FIGURE 4:**
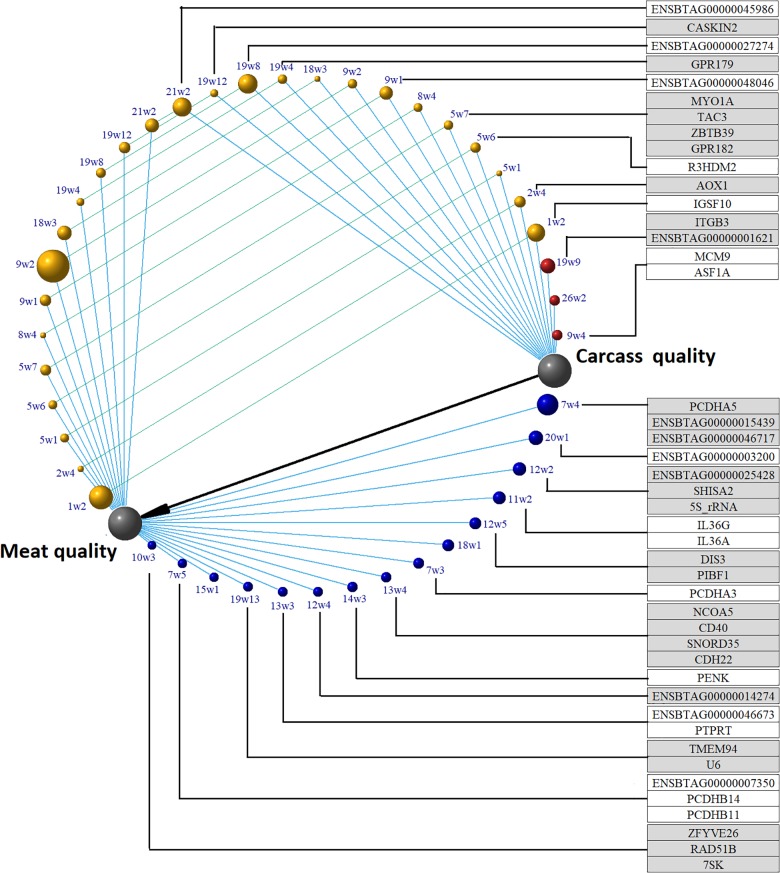
Amount of additive variance explained by 20 SNP-windows, and putative candidate genes underlying latent variables carcass and meat quality in beef cattle. All the phenotypes were measured in *longissimus dorsi*. Only genomic regions that explain more than 0.15% of the additive variance are shown. Gray nodes are the latent phenotypes constructed in the structural analysis. Red nodes are chromosome regions able to explain variability in carcass quality; yellow nodes are chromosome regions able to explain variability in both carcass and meat quality; blue nodes are chromosome regions able to explain variability in meat quality. The black edge shows the causal relationship between carcass and meat quality; blue edges represent the links between chromosome regions and a latent variable; green edges relate the effect of the same chromosomal region on the latent phenotypes carcass quality and meat quality. Red, yellow, and blue node size represents the amount of genetic variance explained by the region. Seven chromosome regions have no annotated genes.

Three chromosome regions harboring four genes were able to explain more than 0.15 of the additive variance present in the carcass quality latent variable (Figure 4 and Supplementary Table S4). There are two important genes inside the 19w9 (BTA19: 47005443–47108805) genomic region: *ENSBTAG00000001621*, the bovine homologous of the human *EF-Hand Calcium Binding Domain 13* (*EFCAB13)*, and *Integrin Subunit Beta 3* (*ITGB3*) which encodes a protein that mediates cell-adhesion to the extra cellular matrix or to other cells through a direct cytoskeletal connection. The ITGB3 protein is a receptor for fibronectin, laminin, prothrombin, matrix metalloproteinase-2, osteomodulin, osteopontin, thrombospondin, and vitronectin. The expression of this gene has been reported as associated with several types of cancer through miRNA mediation, and it was also reported to modulate cell proliferation, migration, and invasion (Shang et al., 2014; Ye et al., 2014; Sun et al., 2015; Wei et al., 2016). Inside the 26w2 region no gene has been reported; however, this genomic region is close to the *Marker of Proliferation Ki-67* (*MKI67*). The biological role of this gene is related to mitotic chromosome dispersion after nuclear envelope disassembly, and MKI67 has a tendency to be highly expressed in obese people as a signal of adipocyte proliferation (Haim et al., 2015^1^). Inside the 9w4 region, the *Minichromosome Maintenance 9 Homologous Recombination Repair Factor* (*MCM9*) and *Anti-Silencing Function 1A Histone Chaperone* (*ASF1A*) genes are located. The former is involved in homologous recombination repair of the DNA. The *ASF1A* gene is a histone chaperone and was shown to facilitate histone deposition, exchange and removal during nucleosome assembly and disassembly.

One gene cluster was identified through DAVID in the 179-gene list associated with carcass quality (Supplementary Table S5). This cluster contains integral components of plasma and organelle membranes, as well as extracellular proteins. The CD320 Molecule (CD320), Cytochrome B561 (CYB561), Galactose-3-*O*-Sulfotransferase 3 (GAL3ST3), Glycerophosphodiester Phosphodiesterase 1 (GDE1), Lysophosphatidic Acid Receptor 1 (LPAR1), Phosphatidylinositol Glycan Anchor Biosynthesis Class S (PIGS), Phosphoinositide-3-Kinase Interacting Protein 1 (PIK3IP1), Prostaglandin I2 Receptor (PTGIR), Rh Associated Glycoprotein (RHAG), Rh Family C Glycoprotein (RHCG), Solute Carrier Family 5 Member 10 (SLC5A10), and Stromal Interaction Molecule 1 (STIM1) are integral plasma or organelle membrane constituents. The Cathepsin H (CTSH), Cathepsin V (CTSL2), Interleukin 7 (IL7), Inter-Alpha-Trypsin Inhibitor Heavy Chain 1 (ITIH1), Interleukin 6 Family Cytokine (LIF), and Oncostatin M (OSM) are extracellular associated proteins. Some of the associated integral membrane proteins are involved in adipocyte organization signaling. Expression of *GDE1, LPAR1*, and *STIM1* has been associated with hepatic triglyceride amount, promotion of fibrosis and adipogenesis inhibition, and adipocyte differentiation, respectively (Graham et al., 2009; Hui et al., 2015; Lacroix et al., 2015).

Two peptidases CTSH and CTSL2 and the peptidase inhibitor ITIH1 are involved in adipocyte differentiation. Similarly, another peptidase, the disintegrin and metalloproteinase domain-containing protein 12 (ADAM12) has been reported as involved in adipocyte differentiation. The ADAM12 can induce cell morphology changes and adipogenic differentiation by degrading the ITGB1 protein in transgenic mice. The ITGB1 degradation induces actin cytoskeleton and extracellular reorganization in adipose tissue since this integrin serves as a cytoskeletal anchoring protein. As a result, these transgenic mice had higher body weight, total body fat mass, abdominal fat mass but they were normoglycemic (Kawaguchi et al., 2002, 2003). Extracellular remodeling and overexpression of cathepsins (CTSA, CTSH, CTSK, CTSL, CTSS, and CTSZ), some types of collagen, matrix metalloproteinases (MMPs), tissue inhibitors of metalloproteinases (TIMP1) and some integrins (ITGAD, ITGAM, and ITGAX) were reported as a consequence of consuming diets with high fat content (Choi et al., 2015).

The LIF and OSM proteins are involved in cellular differentiation and proliferation^1^. Hogan (2005) documented that LIF did not affect adipocyte proliferation but it was able to modify triglyceride accumulation during adipogenesis. On the other hand, adipogenesis can be strongly inhibited by OSM through the RAS/ERK and RAS/signal transducer and activator of transcription 5 (STAT5) signaling pathways; additionally, OSM can promote adipocyte dedifferentiation by decreasing adiponectin expression (Miyaoka et al., 2006; Song et al., 2007).

All the proteins from the cluster mentioned above have a potential role in the adipocyte proliferation and differentiation in subcutaneous fat. In humans, *CD320, CTSH, CTSL2, EFCAB13, IL7, ITGB3, ITIH1, LIF, LPAR1, OSM, PIGS, PIK3IP1*, and *PTGIR* have been reported to have a higher mRNA expression in adipose tissue than in skeletal muscle. This would explain and support the association of these chromosome regions specifically with carcass quality through a larger effect on FOR (subcutaneous fat) and no association with the meat quality latent variable which is mostly driven by MARB.

The control of the cellular proliferation and differentiation begins with the perception of the biochemical and mechanical signals from the extracellular matrix through transmembrane proteins such as integrins, and this information is used to determine cell fate. Some members of the *Integrin Family like Integrin Alpha V* (*ITGAV*), *Integrin Alpha 5* (*ITGA5*) and *Integrin Beta*, and structural proteins such as fibronectin, actin, vinculin, α-actinin, and tropomyosin were reported (Rodríguez Fernández and Ben-Ze’ev, 1989; Liu et al., 2005; Morandi et al., 2016) to be highly expressed in undifferentiated cells but strongly downregulated in mature adipocytes. This would suggest an important role of integrins in adipogenic differentiation. Thus, changes in the expression of cellular microfilaments and cellular transmembrane proteins that communicate the extracellular matrix signal could affect cell adhesion and cell shape during adipocyte differentiation. In support of this hypothesis, Farnier et al. (2003) reported that the Integrin Beta 1 (ITGB1) was more abundant in the plasma membrane of larger adipocytes which have higher fatty acid synthase and lipoprotein lipase activities, GLUT4 protein concentration and leptin expression than small adipocytes. These changes could be caused by the integrin/extracellular signal-regulated kinase (ERK) signaling pathway which is responsible for adipocyte size plasticity.

In the present study the chromosome regions 2w6, 2w7, 16w1, 17w2, 19w3, and 26w1 were associated with carcass quality and have been previously found to be associated with fatty acid composition and WBSF (McClure et al., 2012; Saatchi et al., 2013; Cesar et al., 2014; Chen et al., 2015).

#### Genomic Regions Associated With the Meat Quality Latent Variable

Fifteen chromosome regions harboring 28 genes explained more than 0.15% of the additive genetic variance for the meat quality latent variable (Figure 4 and Supplementary Table S4). The following genes were located inside 7w4: *ENSBTAG00000015439, Protocadherin Alpha 5* (*PCDHA5*) and *ENSBTAG00000046717* genes. The *PCDHA6*, the human homologous of *ENSBTAG00000015439*, is an integral component of plasma membrane involved in cell adhesion, cell–cell signaling, and calcium ion binding. The *PCDHA6* is also involved in the establishment and maintenance of specific neuronal connections, and it probably has an osteogenic function. Additionally, aberrant methylation of this gene has been described in myelofibrosis cases, and one polymorphism of this receptor was associated with bipolar disorder and major depressive disorder (Rodriguez, 2015; Myrtue Nielsen et al., 2017). The *PCDHA5* encodes a calcium-dependent receptor potentially associated with cell-adhesion but very limited information is available on this gene.

The 12w2 chromosome region contained the *Shisa Family Member 2* (*SHISA2*), *ENSBTAG00000025428* and *5S_rRNA* genes. The *SHISA2* encodes an endoplasmic reticulum associated protein, and it is involved in maturation of presomitic mesoderm cells. Overexpression of this protein has been identified in high-grade prostate cancer cells, and its downregulation suppresses cancer cell viability, proliferation and aggressiveness (Tamura et al., 2017). The ENSBTAG00000025428 is a membrane protein involved in phospholipid transport.

The 11w2 region harbors the *Interleukin 36 Gamma* (*IL36G*) and *Interleukin 36 Alpha* (*IL36A*) genes. The protein products of these genes are inflammatory response factors that act on keratinocytes, dendritic cells, and indirectly on T-cells. These factors play an important role in myoblast, gut and skin inflammation, epidermal keratinocyte differentiation and growth (Bachmann et al., 2012; Tortola et al., 2012; Sugiura et al., 2013; Takahashi et al., 2015; Lachner et al., 2017). The 12w5 chromosome region harbors the *DIS3 Homolog, Exosome Endoribonuclease and 3′–5′ Exoribonuclease* (*DIS3*) and *Progesterone Immunomodulatory Binding Factor 1* (*PIBF1*) genes. The former gene is a putative catalytic component of the RNA exosome with exoribonuclease activity, and PIBF1 is a cytoskeletal pericentriolar protein involved in mitotic spindle pole integrity providing a link between microtubule- and actin-based cytoskeleton (Hagarman, 2010). Highly proliferating breast tumor cells have overexpression of PIBF1 mRNA, and the protein is co-localized with γ-tubulin in the microtubule organizing center/centrosome and Golgi apparatus (Lachmann et al., 2004). Balassa et al. (2018) reported that PIBF1 can modulate E-cadherin expression and metalloproteinases activity, thus it can promote cell–cell adhesion, extracellular matrix degradation and tumor invasion.

The *Family with Sequence Similarity 92 Member B* (*FAM92B*) gene was the closest gene to the 18w1 region. This protein has several lipid-binding BAR domains which are involved in membrane shaping and interaction with actin filaments. The 7w3 chromosome region harbored the *Protocadherin Alpha 3* (*PCDHA3*) gene, another member of the protocadherin alpha subfamily. de Lemos et al. (2018) identified a significant copy number variation region associated with myristic fatty acid contend in *longissimus* *thoracis* muscle samples from Nellore animals. Inside this region, the *PCDHA3, PCDHA6, PCDHA10, PCDHA13* genes are located. The *CD40 Molecule* (*CD40), Cadherin 22* (*CDH22*), *Nuclear Receptor Coactivator 5* (*NCOA5*), *Small Nucleolar RNA*, and *C/D Box 35A* (*SNORD35*) are located in the 13w4 region, and *NCOA5* haploinsufficient male mice develop glucose intolerance and hepatocellular carcinoma with lung metastasis and necrosis (Gao et al., 2013). These mice have abnormal cellular architectural organization, enlarged nucleus, vacuolated hepatocytes, and increased lipid deposition which are typical features of hepatic dysplasia and steatosis. Gao et al. (2013) and Laffitte (2017) reported that NCOA5 can act as a transcription factor (e.g., for IL6), promote cell proliferation and modify glucose cellular consumption and lactate production. The CD40 is a transmembrane protein and *CD40* knockout mice have increased weight gain, impaired insulin secretion and glucose intolerance, proinflammatory gene upregulation, liver steatosis and higher gathering of inflammatory cells in adipose tissue than wild type. Additionally, *CD40* is upregulated in adipocytes from obese male mice (Wolf et al., 2014). The CDH22 is a transmembrane glycoprotein involved in cell–cell adhesion. Downregulation and hypermethylation of *CDH22* is related to progression of melanoma, colorectal cancer, and breast cancer due to cells having a higher capacity to break off and metastasize (Piche et al., 2011; Martín-Sánchez et al., 2017).

The *Proenkephalin* (*PENK*) gene located in the 14w3 chromosome region encodes an endogenous opioid peptide secreted by hypothalamus. A chronic fat diet consumption in rats increased *PENK* expression by 20% and increased circulating levels of triglycerides and leptin. The 12w4 region harbored the *Cytochrome C Oxidase Subunit 5B* (*COX5B*) gene (human homologous of *ENSBTAG00000014274*). Arany et al. (2007) documented that the transcriptional coactivator PGC-1β is able to induce the formation of IIX fibers (oxidative and fast-twitch) and promote mitochondrial biogenesis in skeletal muscle. During this fiber type transition, genes related to mitochondrial metabolism, such as *COX5B*, showed an increased expression. The *Tyrosine Phosphatase Receptor T* (*PTPRT*) and *ENSBTAG00000046673* genes are located inside the 13w3 region. Feng et al. (2014) reported that high fat diets did not induce obesity in the *PTPRT* knockout mice and they also did not develop insulin sensitivity, blood glucose and insulin levels dysfunction. However, these mice have lower cholesterol, higher free fatty acids, and changes in blood triglyceride concentration.

Two different possible mechanisms emerge for these chromosome regions having an association solely with the meat quality latent variable. The first mechanism is through a postmortem proteolysis of structural transmembrane anchoring proteins expressed in skeletal muscle, such as CD40, CDH22, PCDHB11, PCDHB12, PCDHB14, PTPRT, SHISA2, and TMEM94 or cytoskeletal proteins such as NCOA5 and PIBF1. The main proteolytic substrates of the μ-calpain-calpastatin system associated with tenderness are desmin and talin (Monsón et al., 2005; Bee et al., 2007) but unknown minor structural proteins can also play a crucial role in this process. Anchoring proteins may be important during tenderization since they allow the attachment between cytoskeletal proteins, plasma and organelle membranes, and extracellular matrix proteins. This mechanism would be important for the observed TD and CT phenotypes. A significant effect of the μ-calpain-calpastatin system on meat quality in the present population was recently reported (Leal-Gutiérrez et al., 2018; Wright et al., 2018). The second mechanism involves a direct effect on intramuscular fat composition through *CD40, ENSBTAG00000025428, NCOA5, PCDHA3, PENK*, and *PTPRT* which are involved in phospholipid, triglycerides and cholesterol metabolism, fatty acid profile, insulin secretion and glucose tolerance. This mechanism would have a direct effect on TD and JC with minimum side effects on MARB. This hypothesis is supported by numerous reports of association between genomic regions identified in the present study (2w3, 3w1, 3w4, 5w4, 6w2, 8w1, 9w3, 13w3, 13w4, 14w3, 14w4, 15w1, 15w2, 18w1, 19w5, and 28w1) and tenderness, WBSF and fatty acid composition in other populations (McClure et al., 2012; Saatchi et al., 2013; Tizioto et al., 2013; Chen et al., 2015; Magalhães et al., 2016; Castro et al., 2017; Zhu et al., 2017).

#### Genomic Regions Associated Simultaneously With Carcass and Meat Quality Latent Variables

A total number of 13 regions harboring 12 genes explained over 0.15% of the additive genetic variance due to meat quality or carcass quality latent variables (Figure 4 and Supplementary Table S4). The 1w2 chromosome region harbored the *Immunoglobulin Superfamily Member 10* (*IGSF10*) gene. Downregulation of this gene is associated with reduced migration of immature GnRH neurons and the IGSF10 intracellular retention results in hypothalamic amenorrhea, delayed puberty, and hypogonadotropic hypogonadism (Howard et al., 2016). The *Aldehyde Oxidase 1* (*AOX1*) gene, located inside the 2w4 region is an adiponectin regulator. Low adiponectin levels have been associated with obesity. Mice fed high fat content diets had higher expression of *AOX1*, reduced adiponectin and developed hepatic steatosis, and *AOX1* knock-down preadipocytes develop impaired triglyceride and adiponectin levels (Neumeier et al., 2006; Weigert et al., 2008).

The 5w1 region did not harbor any gene but *Plexin C1* (*PLXNC1*) is the closest gene to this chromosome region. This gene encodes a transmembrane protein which is a calcium-dependent cell adhesion molecule able to modulate cytoskeletal rearrangement and interleukin secretion. The *R3H Domain Containing 2* (*R3HDM2*) gene is located inside the 5w6 chromosome region. The 5w7 region harbored the genes *G Protein-Coupled Receptor 182* (*GPR182*), *Myosin IA* (*MYO1A*), *Tachykinin 3* (*TAC3*), *Zinc Finger and BTB Domain Containing 39* (*ZBTB39*). Since TAC3 and GPR182 are tissue restricted, the association of 5w7 and 5w6 with both latent variables may be due to the ZBTB39 transcription factor or the MYO1A non-filamentous protein.

No gene was located inside 18w3, but *Cadherin 11* (*CDH11*) was the closest gene to this region; this gene encodes a transmembrane glycoprotein component of adherence junction. The *CDH11* gene is determined as an important factor in skeletal architecture, osteoblast differentiation and osteoid matrix mineralization, and has been reported as a glioma invasion-associated candidate gene (Kawaguchi et al., 2000; Lei et al., 2006; Farber et al., 2011; Delic et al., 2012). Regulation of *CDH11* expression by miRNAs is related to cervical cancer cell progression, aneurysmal bone cyst and osteosarcoma (Nakajima et al., 2008; Deng et al., 2013; Yao et al., 2016). Retinoblastomas in knockout *CDH11* mice have faster tumor growth and decreased cell death than a normal tissue. These features were associated with decreased caspase-3 activation and increased β-catenin expression, showing that CDH11 displays tumor suppressor properties through activation of cell death (Marchong et al., 2010).

These chromosome regions have simultaneous effects on meat and carcass quality indicating a direct effect on both traits. Similar mechanisms to the ones described previously can be hypothesized for these associations. The first mechanism involves changes in the postmortem proteolysis of structural transmembrane anchoring proteins with downstream effect on TD, CT, and JC but an additional effect on cellular compartmentalization in adipocytes resulting in a direct effect on FOR, MARB, and QG. These structural proteins are required for an adequate cellular compartmentalization, which is an important feature for adipocyte size adaptation (Farnier et al., 2003). Related proteins to this mechanism are CAVIN4, CDH11, OGFRL1, PLPPR1, PLXNC1, and TMEFF1. Proteins such as AOX1, CAVIN4, and CASKIN2 may be crucial for intramuscular fat accumulation because they have been found to be involved in energy metabolism contributing to this phenotype through the second mechanisms. Beside these two mechanisms, a third one involves cell proliferation. Proteins such as CASKIN2, CDH11, ENSBTAG00000048046, and OGFRL1 are cell proliferation promoters or cell cycle modulators influencing intramuscular and subcutaneous adipocyte proliferation (MARB and FOR). In this regard, several genomic regions inside or close to 2w4, 8w4, 9w1, 14w2, 19w10, 19w4, and 19w8 are associated with FOR and fatty acid composition (Ishii et al., 2013; Saatchi et al., 2013; Chen et al., 2015; Li et al., 2017; Zhu et al., 2017).

#### Genomic Regions With Effects on Meat Quality Latent Variable Through Carcass Quality

Six chromosome regions explained between 0.1 and 0.15% of the additive genetic present in the constructed latent variable meat quality through an effect on carcass quality (Supplementary Table S4). Genes located inside these regions included *Ataxin 7 like 1* (*ATXN7L1*), *Insulin Receptor Substrate 2* (*IRS2*), *Spectrin Repeat Containing Nuclear Envelope Protein 2* (*SYNE2*), and *YdjC Chitooligosaccharide Deacetylase Homolog* (*YDJC*). The SYNE2 protein is a structural protein with a possible role in proteolysis and cell compartmentalization (Zhang et al., 2007), IRS2 and YDJC are involved in energy metabolism, and ATXN7L1 has been found associated with cancer and cellular proliferation (Poetsch et al., 2011). The effect of these chromosome regions on the meat quality latent variable through carcass quality could be due mainly to an effect on MARB that has an effect on TD and JC.

Genomic regions inside or close to the 4w3, 17w4, 20w2, and 27w1 chromosome regions have been previously reported as associated with marbling, fatty acid composition and WBSF (Cesar et al., 2014; Chen et al., 2015; Castro et al., 2017; Li et al., 2017) suggesting that the effect reported on WBSF may be an indirect effect of these genomic regions on meat quality through a direct effect on MARB.

## Conclusion

A SE model with carcass quality as an independent latent variable and meat quality as a dependent latent variable satisfied the variance-covariance structure present in the sample data. Carcass quality included QG, FOR and MARB, and meat quality was measured by JC, TD, and CT. The whole-genome scan analysis allowed the discrimination between genomic regions with effect on carcass quality and meat quality independently, but also made possible to uncover genomic regions responsible for the genetic correlation between these latent phenotypes.

Thirty one chromosome regions harboring 44 genes explained more than 0.15% of the additive genetic variance present in the carcass and meat quality latent variables. Three regions (harboring four genes) were associated with the carcass quality latent variable, 15 regions (harboring 28 genes) were associated with the meat quality latent variable, and 13 regions (harboring 12 genes) were associated with both meat quality and carcass quality. Six chromosome regions were able to explain between 0.1 and 0.15% of the variability present in the latent variables meat quality on an indirect manner given a direct effect on carcass quality.

Three possible mechanisms can explain the observed association of these chromosome regions with the carcass and meat quality latent variables. The presence of several anchoring and cytoskeletal proteins inside the associated genomic regions identified in the present analysis suggests a possible mechanism involving postmortem proteolysis of structural transmembrane proteins. These structural proteins are also related to cellular compartmentalization in adipocytes, which is a requirement for adipocyte size adaptation. The second mechanism is related to intramuscular fat composition and deposition as suggested by multiple genes involved in phospholipid, triglycerides and cholesterol metabolism, fatty acid profile, insulin secretion, glucose tolerance and fat deposition. The third mechanism involves cell differentiation and proliferation given that several proteins have been reported promoters of adipocyte proliferation.

## Author Contributions

JL-G conducted all analyses and drafted the manuscript. FR assisted with the ssGBLUP analysis and manuscript. ME assisted with the analysis and manuscript. DJ assisted with data collection and manuscript. FP and RM conceived the study and assisted with the data analyses and manuscript preparation.

## Conflict of Interest Statement

The authors declare that the research was conducted in the absence of any commercial or financial relationships that could be construed as a potential conflict of interest.

## Footnotes

1 www.genecards.org

## Supplementary Material

FIGURE S1 Phenotypic correlation matrix for the 19 observed phenotypes utilized to construct the latent variables growth, carcass quality and meat quality in *longissimus dorsi*. BW, birth weight; WW, 205-day adjusted weaning weight; YW, 365-day adjusted yearling weight; SW, 525-day adjusted slaughter weight; ADBW, adjusted daily gain from birth to weaning; ADWY, adjusted daily gain from weaning to yearling; ADYS, adjusted daily gain from yearling to slaughter; HCW, hot carcass weight; DP, dressing percentage; MARB, marbling; FOR, fat over ribeye; KPH, kidney, pelvic and heart fat; REA, ribeye area; YG, yield grade; QG, quality grade; JC, juiciness; FL, flavor; TD, tenderness; CT, connective tissue.

TABLE S1 Variance-covariance matrix of the observed phenotypes utilized to measure the latent variables carcass quality and meat quality in *longissimus dorsi*. MARB, marbling; FOR, fat over ribeye; QG, quality grade; JC, juiciness; TD, tenderness; CT, connective tissue.

TABLE S2 The SE estimations for latent variables, regression and covariances in the final model. The latent variables carcass quality and meat quality were measured by the observed phenotypes: MARB, marbling; FOR, fat over ribeye; YG, yield grade, JC, juiciness; TD, tenderness; CT, connective tissue in *longissimus dorsi* muscle.

TABLE S3 The ssGBLUP for the latent variables carcass quality, uncorrected meat quality and meat quality in *longissimus dorsi* muscle in an Angus-Brahman multibreed population. Position1 and position2 relates the chromosomic location of the 20 adjacent SNP-window region able to explain more than 0.1% of the genetic variability present in the latent variables carcass quality or meat quality. ^∗^The chromosome regions location is shown using the Bos_taurus_UMD_3.1.1 assembly.

TABLE S4 List of genes inside each associated chromosome region of 20 adjacent SNP-window for the latent variables carcass quality and meat quality identified in *longissimus dorsi* from an Angus-Brahman multibreed herd. Each selected region is able to explain more than 0.1% of the additive variance present in at least one latent variable. It is shown the gene stable ID, gene name, gene description, and gene location. Additionally, the type of effect associated with each region is presented.

TABLE S5 Gene clusters identified inside chromosome regions associated with the latent variables carcass quality and meat quality found in *longissimus dorsi* from an Angus-Brahman multibreed herd. These clusters were determined using regions able to explain more than 0.05% of the additive variance present in at least one latent variable. The analysis was performed individually by type of effect.
