# Supplementary figures and images for: Structural Equation Modeling and Whole-Genome Scans Uncover Chromosome Regions and Enriched Pathways for Carcass and Meat Quality in Beef
Source: Front Genet. 2018 Nov 13;9:532. doi: 10.3389/fgene.2018.00532 (PMC6282042; doi:10.3389/fgene.2018.00532)

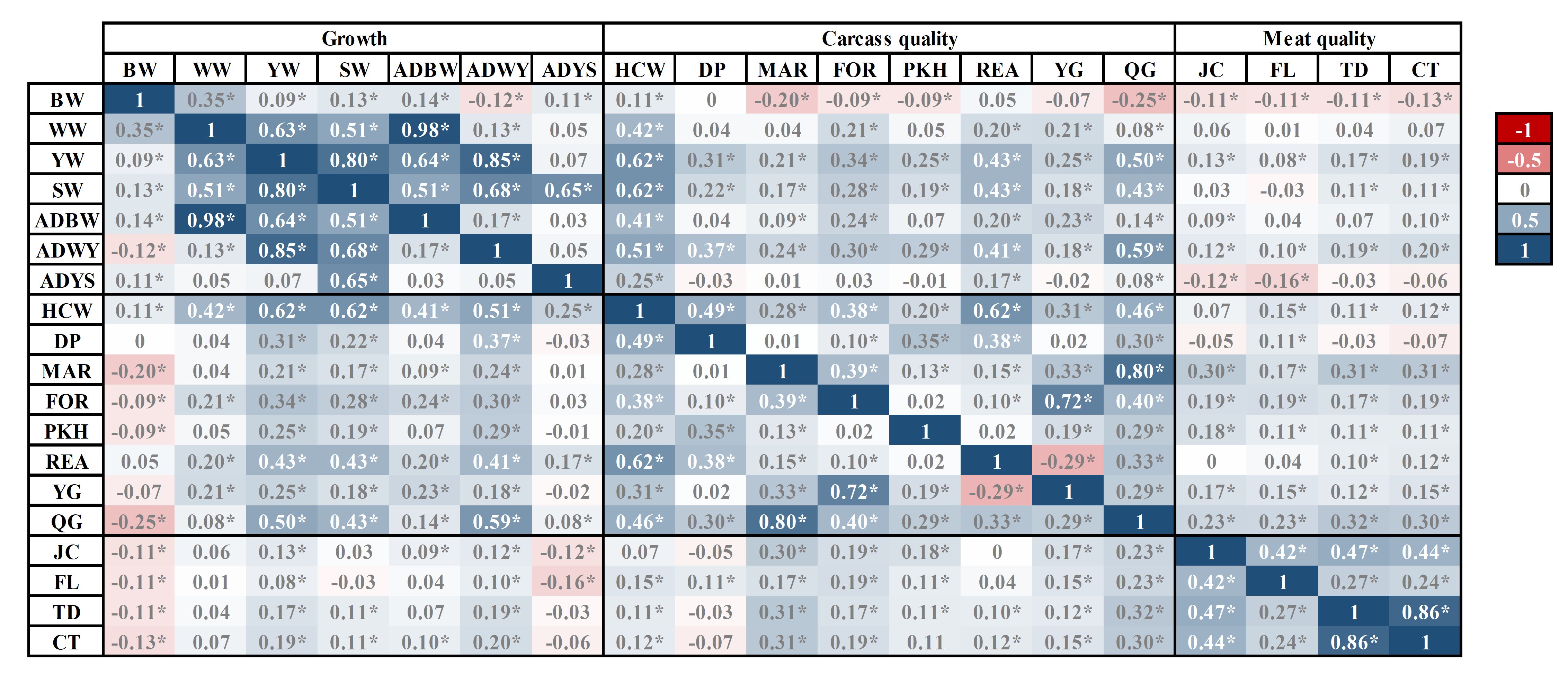

Supplement: FIGURE S1 — Phenotypic correlation matrix for the 19 observed phenotypes utilized to construct the latent variables growth, carcass quality and meat quality in longissimus dorsi. BW, birth weight; WW, 205-day adjusted weaning weight; YW, 365-day adjusted yearling weight; SW, 525-day adjusted slaughter weight; ADBW, adjusted daily gain from birth to weaning; ADWY, adjusted daily gain from weaning to yearling; ADYS, adjusted daily gain from yearling to slaughter; HCW, hot carcass weight; DP, dressing percentage; MARB, marbling; FOR, fat over ribeye; KPH, kidney, pelvic and heart fat; REA, ribeye area; YG, yield grade; QG, quality grade; JC, juiciness; FL, flavor; TD, tenderness; CT, connective tissue. [file Image_1.TIF]
